# Knowledge, Awareness and Practice with Antimicrobial Stewardship Programmes among Healthcare Providers in a Ghanaian Tertiary Hospital

**DOI:** 10.3390/antibiotics11010006

**Published:** 2021-12-22

**Authors:** Eneyi E. Kpokiri, Misha Ladva, Cornelius C. Dodoo, Emmanuel Orman, Thelma Alalbila Aku, Adelaide Mensah, Jonathan Jato, Kwadwo A. Mfoafo, Isaac Folitse, Araba Hutton-Nyameaye, Inemesit Okon-Ben, Paapa Mensah-Kane, Emmanuel Sarkodie, Benedict Awadzi, Yogini H. Jani

**Affiliations:** 1 Department of Clinical Research, Faculty of Infectious and Tropical Diseases, London School of Hygiene and Tropical Medicine, London WC1E 7HT, UK; 2 Joint Research Office, University College London, London W1T 7BN, UK; misha.ladva@ucl.ac.uk; 3 School of Pharmacy, University of Health and Allied Sciences, PMB 31 Ho, Ghana; cdodoo@uhas.edu.gh (C.C.D.); eorman@uhas.edu.gh (E.O.); talalbila@uhas.edu.gh (T.A.A.); amensah@uhas.edu.gh (A.M.); jjato@uhas.edu.gh (J.J.); kmfoafo@uhas.edu.gh (K.A.M.); aharaba@uhas.edu.gh (A.H.-N.); ioben@uhas.edu.gh (I.O.-B.); pmensah-kane@uhas.edu.gh (P.M.-K.); 4 Ho Teaching Hospital, P.O. Box, MA 374 Ho, Ghana; ifolitse@yahoo.com (I.F.); benedictawadzi@gmail.com (B.A.); 5 Kwame Nkrumah University of Science and Technology Hospital, MCPG 988 Kumasi, Ghana; sakem2k5@yahoo.com; 6 Centre for Medicines Optimisation Research and Education, University College London Hospitals, NHS Foundation Trust, London NW1 2BU, UK; yogini.jani@nhs.net

**Keywords:** antimicrobial resistance, antimicrobial stewardship, healthcare providers, LMICs

## Abstract

Antimicrobial resistance (AMR) is a significant problem in global health today, particularly in low- and middle-income countries (LMICs) where antimicrobial stewardship programmes are yet to be successfully implemented. We established a partnership between AMR pharmacists from a UK NHS hospital and in Ho Teaching Hospital with the aim of enhancing antimicrobial stewardship knowledge and practice among healthcare providers through an educational intervention. We employed a mixed-method approach that included an initial survey on knowledge and awareness before and after training, followed by qualitative interviews with healthcare providers conducted six months after delivery of training. This study was carried out in two phases in Ho Teaching Hospital with healthcare professionals, including pharmacists, medical doctors, nurses and medical laboratory scientists. Ethical approval was obtained prior to data collection. In the first phase, we surveyed 50 healthcare providers, including nurses (33%), pharmacists (29%) and biomedical scientists (23%). Of these, 58% of participants had engaged in continuous professional development on AMR/AMS, and above 95% demonstrated good knowledge on the general use of antibiotics. A total of 18 participants, which included four medical doctors, five pharmacists, four nurses, two midwives and three biomedical scientists, were interviewed in the second phase and demonstrated greater awareness of AMS practices, particularly the role of education for patients, as well as healthcare professionals. We found that knowledge and practice with AMS was markedly improved six months after the training session. There is limited practice of AMS in LMICs; however, through AMR-focused training, we demonstrated improved AMS skills and practice among healthcare providers in Ho Teaching Hospital. There is a need for continuous AMR training sessions for healthcare professionals in resource-limited settings.

## 1. Introduction

Antimicrobial resistance (AMR) is a significant problem in global health today. It is severe in low- and middle-income countries (LMICs), where the burden of infectious diseases is much higher. There is limited information on the extent of this problem in these settings. Global mortality projections due to AMR in the next few decades show that the greatest impact will be in Africa and Asia [1]. Inappropriate prescription and use of antibiotics have been shown to contribute significantly to this problem. Antimicrobial stewardship programmes (AMS) are designed to improve the use of antimicrobials, and they have been developed and implemented successfully with corresponding impacts in most high-income settings. In 2015, the WHO released a Global Action Plan with a combination of AMS interventions, and it is yet to be fully implemented and tested in most LMICs due to the lack of required resources [2]. There is an urgent need to identify practical strategies for supporting the delivery of AMS in these settings.

Widespread AMR has been documented in Ghana in national reports and published research [3]. Ghana’s first national action plan for AMR, spanning from 2017 to 2021, was published in 2017 by the Ghanaian Ministry of Health with the support of the WHO and the Food and Agriculture Organization of the United Nations. Multiple-drug resistance is as high as 78%, and resistance to common antimicrobials such as tetracycline, cotrimoxazole and ampicillin is around 70%. High prevalence of methicillin-resistant *Staphylococcus aureus* (MRSA) has also been documented [3]. Ghana’s national action plan outlines strategies for implementing stewardship to include monitoring and surveillance, education, training and provision of guidelines [4]. Reports from Ghana have highlighted gaps in optimal antimicrobial use, even in hospitals [5]. There is a need to optimise antibiotic prescription practices and strengthen monitoring, surveillance and documentation of antibiotic use and related processes. There is a paucity of evidence on the knowledge and extent to which healthcare staff in Ghana are involved with antimicrobial stewardship programmes. Earlier reports documenting inappropriate use of antimicrobials in Ghana had also called for educational programmes on antibiotic use [6,7].

The Tropical Health and Education Trust (THET), together with the Commonwealth Partnerships for Antimicrobial Stewardship (CwPAMS) developed and funded a partnership model between United Kingdom (UK) National Health Service (NHS) hospitals and African partners [8]. The goal of the partnerships was to leverage the expertise of the NHS to address AMR challenges with the input and resources of the African partners. This paper reports part of the activities and results of one of the partnerships of the University College London Hospital NHS foundation trust (UCLH) with the University of Health and Allied Sciences (UHAS) and Ho Teaching Hospital, which are both in the Ho municipality in the Volta region of Ghana. The activities carried out in this partnership included a periodic Global Point Prevalence Survey (GPPS) [9] to establish patterns of antimicrobial use and an AMR/AMS training and capacity-building workshop for healthcare professionals. The aim of this study was to assess the improvement in AMS knowledge after a training session and to build the capacity and skills of healthcare professionals in order to support the implementation of antimicrobial stewardship programmes in Ho Teaching Hospital in the Volta region of Ghana.

## 2. Methods

### 2.1. Study Setting

This study was conducted in Ho Teaching Hospital, which is located in the Volta region of Ghana. The hospital is a tertiary care hospital and serves as a teaching hospital with a staff strength of about 1200, 306 bed spaces and 14 wards. Ghana is low- and middle-income country located in the sub-Saharan part of West Africa. Ghana has an established National Health Insurance Scheme (NHIS) that provides a broad range of healthcare services to Ghanaians through district, mutual and private health insurance schemes [10].

### 2.2. Study Design

We employed a bottom-up approach with a mixed-method study design [11,12]. This included an initial survey of healthcare providers’ knowledge, awareness and practice (KAP) with respect to antimicrobial resistance and AMS just before and after the delivery of an educational intervention (Box 1). This was then followed with semi-structured interviews six months after the training to assess changes in AMR/AMS knowledge and awareness, as well as improvements in practice. Partners from UCLH with expertise in AMR practice and research developed and delivered AMR-/AMS- and quality-improvement-focused training over a 3-day period.

Box 1 **The educational intervention and activities.**

Topics included in the AMR/AMS Training ProgrammeAMR/AMS—Antimicrobial Resistance/Antimicrobial StewardshipAwaRE—Access Watch, REserveWHO—World Health Organisation
Training block 1
AMR/AMS—definitions; global concern, WHO position, United Kingdom’s and Ghana’s positionsAWaRE lists and what they meanExamples of AMS activities—using specific case scenarios and different infrastructures, e.g., paper prescribing systems vs. electronic systemsGlobal Point Prevalence SurveyIntroduction to Quality Improvement and Behavioural Change theories

Training block 2
Infection prevention and controlFeedback and review of results from GPPS 1Consider areas for change; apply QI methodology and Behavioural Change theoriesConduct Global Point Prevalence Survey 2

Training block 3
Enabling pharmacists as AMS leaders and champions—role of clinical pharmacists/advanced pharmacy practiceFeedback and review of results from Global Point Prevalence Survey 2Reiterate QI approaches and Behavioural Change theoriesConduct Global Point Prevalence Survey 3

### 2.3. Data Collection

For phase 1, we designed a data collection tool (Supplemental File S1) to retrieve data on knowledge, awareness and practice of AMR/AMS among 50 healthcare providers. For phase 2, an interview topic guide was designed (Supplemental File S2) and used to conduct interviews with 18 healthcare providers. Prior to the interviews, the participants were provided with an invitation and information leaflet with relevant information about the study as an invitation to participate. Participants were also provided with a consent form to sign, and all interviews were audio recorded.

### 2.4. Data Analysis

**Figure 1:**
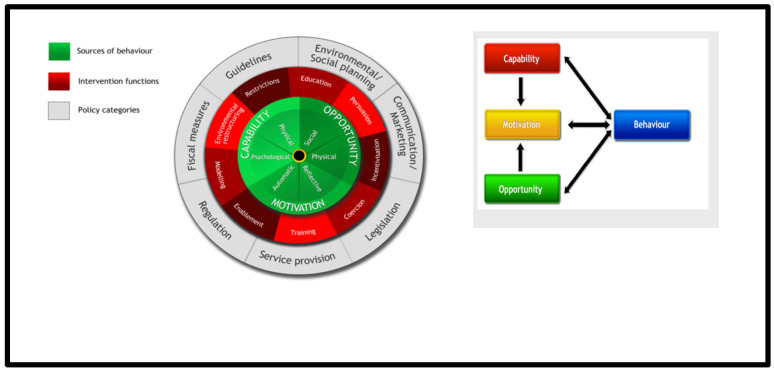
The Behaviour Change Wheel and COM-B model, adapted from ref. [13].

Data collected from the initial pre- and post-training surveys were entered into and analysed using the IBM Statistical Package for Social Sciences (SPSS) version 22. Results were presented using basic descriptive frequencies. All interviews were transcribed verbatim, and transcripts were read repeatedly and then coded individually to identify similarities and differences. All transcripts were coded and reviewed alongside the generation of a coding frame. Codes were also mapped unto the Behaviour Change Wheel (BCW) framework based on the influence of the model of Capability, Opportunity and Motivation on Behavioural outcomes (COM-B) (see Figure 1 below) [13]. Similar codes were grouped for themes and high-level findings, which were presented as a narrative using quotes to support the main themes. To ensure validity, all transcripts were coded by the researcher and randomly validated by another team member (Supplementary Files S3 and S4).

### 2.5. Ethical Approval

Ethical approval was obtained via an ethics application made to the research and ethics committee of the University of Health and Allied Sciences. Ethics reference number: UHAS-REC A.9 [4] 18–19 (1 July 2019)

## 3. Results

Phase 1: AMR knowledge awareness and practice before and after an AMR/AMS training session.

### 3.1. Socio-Demographics

A total of 50 healthcare professionals attended the AMR training session, including nurses (33%), pharmacists (29%) and medical laboratory scientists (23%) as shown in Table 1. Of these, 96% were aged between 25 and 44 years, with slightly more females (54%) than males (46%). Most participants (96%) had achieved a level education of up to a master’s degree.

### 3.2. Knowledge of AMR/AMS Terminology

Up to 58% of the participants engaged in CPD with topics on AMR/AMS, and above 95% demonstrated good knowledge on the general use of antibiotics. While many were familiar with terms such as antibiotic resistance and antimicrobial resistance, more than half were not conversant with antimicrobial stewardship, superbugs and AMR capacity building. However, the proportions demonstrating good knowledge of AMR/AMS increased after the training (Table 2).

### 3.3. General Knowledge about Antibiotics

The participants demonstrated good knowledge of antibiotic use, which further improved after the training sessions. The majority of the participants either strongly agreed or agreed with the statements on antibiotic use (Table 3).

### 3.4. AMR Awareness among Health Professionals

Knowledge about AMR was low among the HCPs. Less than 50% of the respondents demonstrated good AMR knowledge in some of the questions, and this improved considerably after training (Table 4).

### 3.5. AMR Practice

More than 80% of the participants agreed that patients’ clinical conditions and microbiology results were key determinants for antibiotic prescriptions, while more than 97% agreed that there was a lack of effective diagnostic tools and that inappropriate prescriptions of antibiotics still exist. About half of the participants confirmed that there were no specific prescription policies and protocols and that the antibiotics available in the facility were unable to treat some infections. The national Standard Treatment Guideline (STG) is the most consulted reference when prescribing antibiotics, followed by the British National Formulary (BNF). Over 90% of the participants agreed that obtaining local antibiotic resistance profiles and changing the attitudes of prescribers and patients will reduce unnecessary antibiotic usage.

#### 3.5.1. Phase 2: Interviews with Healthcare Providers in the HTH

##### Participant Characteristics

For the second phase, we interviewed a total of 18 participants, which included four medical doctors, five pharmacists, four nurses, two midwives and three biomedical scientists. Among the participants were 10 males and eight females, with ages ranging from 27 to 56 years.

##### Knowledge/Awareness of Antimicrobial Resistance Post-Training

The majority of the interviewed participants confirmed that the training on AMR/AMS was very helpful and increased their understanding of AMR. This, in turn, led to changes in their practice, such as reduced empirical prescription of antibiotics and more detailed counselling for patients on antibiotic use.

##### Healthcare Providers’ Roles

The healthcare providers across different specialties explained that they felt more confident carrying out AMS roles after the training. The nurses were competent in antibiotic drug administration and acted as patient advocates to question the antibiotics being prescribed. The pharmacists were also competent in delivering AMS roles and providing information on medications for the rational prescription of antibiotics. Both nurses and pharmacists could support antibiotic prescription decisions, especially when they involve junior doctors.

##### Current Situation with Antibiotic Use in Ghana

The interviewed participants reported general overuse of antibiotics in Ghana with highly empirical and broad-spectrum prescribing. Factors that contribute to this observed practice with antibiotics include a lack of continuous training for prescribers and other healthcare providers (HCPs), poor compliance with guidelines, limited diagnostic services, staff shortages, pressure from patients and the influence of drug companies. To date, there have been no interventions put in place to improve antibiotic use in the hospital in this study.

##### Prescription Patterns and the Decision-Making Process

Commonly prescribed antibiotics include cephalosporins, penicillin, metronidazole, ciprofloxacin, erythromycin, amoxiclav and cefazolin. All of these antibiotics are in the list of essential medicines and are available in hospital formularies in both branded and generic forms. The common indications requiring antibiotic prescriptions include urinary tract infections (UTIs), respiratory tract infections, gastrointestinal tract (GIT) infections, sepsis, sexually transmitted infections (STIs) and surgical prophylaxis. There are standard treatment guidelines; however, the disease type, cost, drug availability and severity of infection are all factors that affect the choice of the antibiotic drug being prescribed.

Recommended strategies for improving antibiotic use and the barriers in implementing these AMS interventions

The respondents highlighted some recommendations for improving antibiotic use, such as including education and training for prescribers, improved labs for microbe-specific treatment, purchase of lab items for testing, antibiotic use checks/audits, employment of more staff to build the workforce and development of local policies. The main barriers to this will be failure to enforce laws and limited funding in healthcare.

##### Sources of Behaviour

From the interview data, the current AMR behaviours that we saw, such as broad-spectrum and highly empirical prescription of antibiotics, fall on inherent local factors, including the lack of continuous training, limited laboratory services and lack of local policies. In order to move forward with the desired behaviours for AMS support and practice, we need to leverage the relevant intervention functions shown on the Behaviour Change Wheel. These include training, enablement, persuasion and environmental restructuring. These functions map to the policy categories of social planning/service provision, guidelines and regulations (Table 5). Some additional results based on the COM-B model are presented below.

*Capability*: HCPs felt more confident and empowered in contributing to AMR topics during rounds and better educating patients after the training.

*Opportunity:* More staff can be employed to create opportunities to deliver additional AMS activities.

*Motivation:* The training was educative, increased knowledge about antibiotic use and helped to improve the AMS practice.

## 4. Discussion

We were able to assess baseline knowledge of antibiotic use and AMR among health professionals in the study hospital. We were also able to demonstrate the impact of an educational intervention on knowledge and awareness of AMR. The healthcare providers described the training as educative and helpful, and they found it useful in their practice with antibiotics. The training generally increased knowledge about antibiotic use and AMR/AMS, and it led to a better understanding of different aspects of antibiotics use, including the importance of proper patient education. After the training, some self-reported changes in practice in the hospital included a reduction in antibiotic use and empirical prescription, as well as better patient counselling and education on antibiotic use. AMR training and related educational programmes for HCPs have been previously employed in different settings to improve the use of antimicrobials [14,15]. In addition to improved knowledge and changes in practices, the HCPs confirmed that they felt more confident in contributing to AMR topics during rounds. The GPPS that was conducted made the prescribers more aware of their prescribing with regard to antibiotics. There was reduced empirical and broad-spectrum antibiotic prescribing. Similar findings have been reported in Saudi Arabia, where comprehensive training and education of HCPs enabled them to implement and deliver AMS services more confidently [16]. However, the majority of the HCPs that attended the earlier training vaguely remembered the WHO’s classification of antibiotics, and a few were still unsure, indicating that continuous AMR training should be organised.

Effective antimicrobial stewardship delivery in hospitals requires a multidisciplinary team effort that includes the medical doctors, who mostly prescribe, pharmacists, nurses, microbiology teams and others. Our training included these different groups of HCPs. Pharmacists are the custodians of drugs within the hospital and can ensure safe and effective use of antibiotics by providing information to other HCPs, as well as counselling patients. Nurses are competent in antibiotic drug administration and act as patient advocates to question the antibiotics being prescribed. While there were guidelines and consultations with other colleagues, the HCPs confirmed that other resources, such as improved laboratory services and an expanded workforce, would increase efficiency in the delivery of AMS roles. The HCPs recommended protocols and guidelines, more AMR training courses, improved laboratory and diagnostic services and availability of patient educational materials in order to support the delivery of AMS in the local hospital. All of these recommendations have also been identified in earlier studies as effective strategies for long-term sustenance of antibiotic stewardship programmes in LMICs [17,18,19].

The HCPs perceived that there is generally irrational overuse of antibiotics in Ghanaian hospitals, citing the broad-spectrum and empirical prescription of antibiotics. Factors contributing to the irrational use of antibiotics in Ghana include a lack of continuous AMR training, poor AMR knowledge, limited diagnostic services, understaffing and pressure from patients and pharmaceutical companies. This is consistent with other findings reported in Ghana [3,20,21].

Broad-spectrum antibiotics, including penicillin, cephalosporins, aminoglycosides and metronidazole, are commonly prescribed. Common indications for which antibiotics are prescribed include respiratory tract infections, UTIs, STIs and GIT infections. Others include typhoid and surgical prophylaxis. In the study hospital, antibiotics are prescribed in both branded and generic forms, but mostly as generics, and they are in the EML and hospital formulary. Similar indications for antibiotic use have previously been identified in Ghana [6,9,22,23].

Presently, there are standard treatment guidelines in the hospital to guide antibiotic use. Most HCPs employ the use of guidelines as often as they need to when dispensing, prescribing or administering antibiotics. While patients can request antibiotics, they have no influence on the decisions on if they eventually get an antibiotic or which antibiotic should be prescribed for and dispensed to them. In addition to laboratory diagnostic reports, the following factors are considered in choosing antibiotics for a patient: the disease and its severity, the cost of the antibiotic and the availability.

Our study had several limitations. First, only a small subset of HCPs from the study hospital were involved in the AMR training programme. The impact of the training would be more effective if a larger cohort had been trained. Secondly, while improvements to knowledge and awareness was assessed immediately after the training, we relied on self-reported changes in practice to assess the impact. This assessment may be subject to reporter bias [24]. However, a key strength of our approach was training those directly involved with antibiotic use within the study hospital, who could then lead a stewardship committee and champion the cause of rational antibiotic use.

The findings of this work have several implications for practice and policies. Given the factors underpinning current antimicrobial use patterns, there is a need for dedicated AMS staff to promote and oversee AMS activities in the hospital. Routine training and AMR-focused workshops should be organised for healthcare providers to reinforce and sustain AMS practices. According to data from the GPPS results, local AMS policies should be developed to guide antimicrobial use in the local hospital context. National and international treatment guidelines should be adapted and tailored for relevance and use in the local settings. There is a need for further research to evaluate the impact of the AMS team on practice and to identify further parameters for long-term sustainability.

## 5. Conclusions

AMR is still a budding public health challenge across the globe, and especially in low-income settings. AMS practice is still in infancy in these settings. We demonstrate how expertise from developed and advanced settings can be leveraged to move AMS practice forward. The findings from this study show that continuous AMR training for healthcare professionals can increase knowledge and awareness, spur behavioural change to improve practice and build momentum for sustainable AMS programmes.

## Figures and Tables

**Table 1:** Socio-demographics of the participants.

Characteristics (*n* = 50)	N (%)
Gender	
Male	23 (46)
Female	27 (55)
Age (years)	
25–34	33 (66)
35–44	15 (30)
45–54	1 (2.0)
55–64	1 (2.0)
Occupation	
Nurse	16 (33.3)
Pharmacist	14 (29.2)
Biomedical Scientist	11 (22.9)
Medical Doctor	3 (6.3)
Pharmacy Technologist	1 (2.08)
Midwife	1 (2.08)
Lecturer	1 (2.08)
Human Resource Manager	1 (2.08)

**Table 2:** Familiarity with AMR/AMS terminology.

Terminology (*n* = 50)	Proportion of Participants	
	Before Training (%)	After Training (%)
Antibiotic resistance	100	100
Superbugs	35	76
Antimicrobial resistance	94	100
Antimicrobial stewardship	54	100
Drug resistance	98	100
*Antimicrobial capacity building*	2	100

**Table 3:** General knowledge about antibiotics.

				Proportion of Participants (%)			
Sr No	Indicator (*n* = 50)		Strongly Agree	Agree	Not Sure	Disagree	Strongly Disagree
1	Antibiotics are used in the management of all infections	Before	22	18	0	26	34
		After	5	7	0	25	63
2	Antibiotic use should be strictly controlled	Before	84	12	0	0	2
		After	83	12	0	0	5
3	Treatment with antibiotics should be stopped once you feel better, especially the expensive ones	Before	2	0	2	24	72
		After	13	0	0	18	70
4	It is okay to use antibiotics that were given to a friend or family member, as long as they were used to treat the same illness	Before	0	0	0	10	90
		After	5	0	0	13	83
5	It is okay to buy the same antibiotics, or request these from a doctor, if you are sick and they helped you get better when you had the same symptoms before	Before	0	0	4	21	74
		After	5	0	0	18	77
6	Frequent use of antibiotics may decrease the efficacy of treatment	Before	56	26	4	6	6
		After	58	23	3	3	15
7	Poor counselling of patients can lead to antibiotic misuse	Before	80	20	0	0	0
		After	90	10	0	0	0
8	Poor skills and knowledge of prescribers can cause irrational antibiotic prescribing	Before	72	26	0	0	2
		After	90	10	0	0	0
9	Patient self-medication can increase AMR	Before	74	24	2	0	0
		After	85	15	0	0	0
10	Inadequate supervision of the administration of medicine	Before	60	36	4	0	0
		After	77	23	0	0	0
11	It is possible for the antibiotics we are using today to stop working properly in the future	Before	58	30	2	4	6
		After	75	23	3	0	0

**Table 4:** Awareness of health professionals of AMR.

Sr No	Indicator		Proportion of Participants (%)				
			Strongly Agree	Agree	Not Sure	Disagree	Strongly Disagree
1	Antibiotic resistance occurs when your body becomes resistant to antibiotics, and they no longer work as well	Before	42	20	4	12	18
		After	45	10	3	23	20
2	Antibiotic resistance is an issue in other countries, but not here	Before	0	2	6	24	68
		After	2	2	0	17	78
3	Antibiotic resistance is an issue that could affect me or my family	Before	76	24	0	0	0
		After	85	15	0	0	0
4	Antibiotic resistance is only a problem for people who take antibiotics regularly	Before	4	12	8	28	48
		After	5	5	0	30	60
5	Antibiotic-resistant infections could make medical procedures such as surgery, organ transplants and cancer treatment much more dangerous	Before	58	30	6	2	4
		After	88	10	0	0	2
6	Bacteria that are resistant to antibiotics can be spread from person to person	Before	30	34	4	12	16
		After	54	32	2	7	5
7	Many infections are becoming increasingly resistant to treatment by antibiotics	Before	54	42	2	2	0
		After	68	27	2	2	0
8	If bacteria are resistant to antibiotics, it can be very difficult or impossible to treat the infections they cause	Before	72	24	0	4	0
		After	73	24	0	2	0
9	Inappropriate use of antibiotics can lead to antibiotic resistance	Before	72	26	0	0	2
		After	90	7	0	0	2
10	Inappropriate use of antibiotics can lead to increased adverse effects and additional burden	Before	56	32	6	4	2
		After	76	22	0	2	0

**Table 5:** Mapping codes and themes to the BCW and COM-B model.

*Themes*	*BCW Framework*		*Sources of Behaviour* *(COM-B)*
	*Intervention Functions*	*Policy Category*	
***Factors contributing to irrational use of antibiotics include***			
Lack of continuous AMR training	*Training*	*Social planning*	*Psychological capabilities*
Poor AMR knowledge	*Enablement*		*Psychological capabilities*
Limited diagnostic services		*Service provision*	*Physical capabilities*
Staff shortages		*Service provision*	
Pressure from patients	*Persuasion/Coercion*		*Reflective motivation*
Incentives from pharmaceutical companies	*Incentivisation*	*Communication/marketing*	
***Recommended strategies to improve antibiotic use***			
Education to increase awareness	*Education/Enablement*		*Automatic motivation*
Checks/audits/monitoring antibiotic use		*Regulations*	
Upgrade laboratory services/purchase lab items for testing	*Environmental restructuring*		*Physical opportunity*
Policies	*Enablement*	*Guidelines*	*Automatic motivation*
Training for prescribers	*Training/Enablement*		*Automatic motivation*
Employ more staff to build workforce	*Environmental restructuring*	*Social planning/Service provision*	*Physical opportunity*
***Barriers to implementing these AMS intervention***			
Lack of funding in healthcare		*Fiscal measures*	
Staff shortages			
Failure to enforce laws		*Regulation/Legislation*	

AMR—Antimicrobial Resistance; *BCW*—Behaviour Change Wheel; *COM-B*—Capability, Opportunity and Motivation on Behaviours.

## Data Availability

The data presented in this study are available on request from the authors.

## Supplementary Materials

The following are available online at https://www.mdpi.com/article/10.3390/antibiotics11010006/s1, Supplementary File S1: Survey Questionnaire for Antibiotic Use and Antimicrobial Resistance; Supplementary File S2: Healthcare Providers’ Interview Topic Guide; Supplementary File S3: AMS Interview Data Analysis; Supplementary File S4: Sample Transcript Coding Frame.
